# Physical Activity and Attitudes Toward Social Media Use of Active Individuals During the COVID-19 Pandemic in Saudi Arabia: Cross-Sectional Survey

**DOI:** 10.3389/fpsyg.2021.707921

**Published:** 2021-08-13

**Authors:** Mezna A. AlMarzooqi

**Affiliations:** Department of Community Health Sciences, College of Applied Medical Sciences, King Saud University, Riyadh, Saudi Arabia

**Keywords:** active individual, physical activity, COVID-19, Saudi Arabia, social media

## Abstract

**Background:** Social media became an integral part of the lives of people because it encourages social relations and shares interests, activities, and real-life connections. As quarantine and lockdown orders are prolonged, many people, as well as those physically active individuals, typically responded to this stressful condition by using social media platforms.

**Objective:** This study aimed to examine the level of physical activity of physically active individuals and their attitudes toward social media use during the COVID-19 pandemic.

**Methods:** A descriptive cross-sectional survey was conducted among physically active individuals in Saudi Arabia between June 2020 and July 2020. An online survey was employed among eligible participants who completed a self-administered questionnaire that covered reasons for physical activity and attitudes toward social media platforms during the COVID-19 pandemic.

**Results:** Of these 323 participants, 29.1% were in the age group of 18–24 years, 66.6% were women, and 67.8% were single. The proportion of participants whose metabolic equivalent of tasks-min/week from vigorous activity <1,680 was 31.9%, and all of the participants follow people or pages in social media related to sports teams and fitness models. The average number of hours spent on social media per day was 2.95 ± 0.90 h. The majority of the participants showed positive attitudes toward social media used for exercise and physical activity. Of the eight variables, age, level of physical activity, and the average of hours spent on social media emerged as significant predictors of attitudes toward the use of social media (*P* < 0.05).

**Conclusions:** The present survey results indicate adverse consequences of home quarantine as reflected by a small proportion of participants who had differences in levels of vigorous activities during the COVID-19 pandemic in Saudi Arabia. Social media appears to be a key avenue to promote and disseminate health interventions to promote physical activity during this COVID-19 pandemic.

## Introduction

The present COVID-19 pandemic has caused a massive unprecedented health crisis (Bavel et al., 2020). The impact of the disease led to different restrictions imposed by different governments to contain the spread of COVID-19. These included quarantines, lockdown, closing of establishments (e.g., restaurants), curfew, restriction of public gathering, and social distancing. The restrictions and rules enforced by the authorities limit also all levels of sports activities and exercises. All these strategies were taken to control the spread of COVID-19. As a consequence, these preventive measures forced people to stay at home, which may lead to sedentary lifestyles or unhealthy habits (Jiménez-Pavón et al., 2020; Kaur et al., 2020).

The emergence of the COVID-19 negatively impacts the daily physical activity of the population. A previous study has shown significant changes in the physical activity behavior in both active and inactive individuals in Canada and the United Kingdom (Lesser and Nienhuis, 2020; Rogers et al., 2020). According to the guidelines of the WHO, adults should engage in at least 300 min of aerobic activity or more than 150 min of vigorous activity per week (World Health Organization, 2020). This situation is extremely worrying because physical inactivity appears to be a major factor in the prevalence of different chronic diseases (Casas et al., 2014; Tavakol et al., 2021). The benefits of physical activity and exercise have been demonstrated to have a significant effect on weight maintenance, improved cardiovascular health, and reduced stress and anxiety (Shahidi et al., 2020). Because of the COVID-19 pandemic, all individuals are struggling in maintaining their normal physical activity intensity level, particularly those physically active individuals.

According to Araújo and Scharhag, physically active individuals or active athletes are people who: actively participate in sports competitions such as marathon; do training to improve their performance; are a member of a local, regional, or national sports association; and devote several of hours for exercise and training and physical activities in all or most days (Araújo and Scharhag, 2016). Having these concepts in mind, to those physically active individuals, the opportunities to keep physically fit have been substantially diminished. Home training programs have been used as an alternative in maintaining their physical condition. Coaches of elite athletes have formulated specific recommendations for home training programs and used new media technologies such as social media platforms to communicate, monitor, and assist their athletes (Hayes, 2020; Jukic et al., 2020; Toresdahl and Asif, 2020; Yousfi et al., 2020).

Social media became an integral part of the lives of people because it encourages social relations and shares interests, activities, and real-life connections (Shaheen et al., 2020). It considers one of the most important factors that can affect positively or negatively the health and behavior of people. As quarantine and lockdown orders are prolonged, many people, as well as those physically active individuals, typically responded to this stressful condition by using social media platforms (Yousfi et al., 2020). A recent report in the United States, the United Kingdom, and Australia shows a dramatic increase in online searches for topics about exercise (Ding et al., 2020; To et al., 2021). In addition, fitness influencers offered live training sessions to promote physical activities during the COVID-19 lockdown (Godefroy, 2020). However, engaging in physical activity has been a major challenge during this period even if there is an option of home training programs. The attitudes and behavior of the people toward physical activity during this time varied from country to country. For example, a recent study that reported home quarantine resulted in a 28% increase or 5–8 h of sitting time and decreased levels of all physical activities (Ammar et al., 2020). Meanwhile, data from a fitness app “WHOOP” platform showed a 1.1% increase in exercise frequency and almost 2% increase in time spent on a high level of intensity exercise (Capodilupo and Miller, 2020). The inconsistencies in the reported findings may indicate that the impact of the COVID-19 pandemic and implemented preventive measures varied among population groups with different demographic characteristics. Additionally, there is limited evidence that evaluates the attitude of physical activity of those individuals who were affected by these restrictions in Saud Arabia.

While the future of COVID-19 is still unknown, there is a need to investigate how people coped when facing the COVID-19 pandemic. Also, there are growing concerns about the effect of lockdown and quarantine that has placed limitations on those physically active individuals. In addition, in an attempt to add evidence to the existing studies about behaviors of physically active individuals, we explore their attitudes toward using social media during this period, since this is the most prominent communication tool among this population. In particular, this study aimed to examine (1) the level of physical activity of physically active individuals, (2) their attitudes toward social media use during this COVID-19 pandemic, and (3) to determine the association of attitudes of physically active individuals toward social media with their demographic characteristics.

## Methods

### Study Design and Participants

This descriptive cross-sectional study was conducted from June 2020 to July 2020. A total of 323 physically active individuals who were living in Saudi Arabia during the COVID-19 pandemic participated in this study. All physically active individuals, a member of a sports club exercising equal to or more than 150–300 min of moderate activity a week, were deemed eligible to participate in this study. The design and protocol of this study were approved and provided by the King Saud University Ethics Research Committee. Informed consent was obtained before participating in this study. All consented participants received an invitation including a link to an online survey and participant information through a sports club group.

### Instrument

Data were collected in three parts using three assessment tools: (1) the demographic section that consists of age, sex, marital status, educational level, and body mass index (BMI), (2) and the International Physical Activity Questionnaire (IPAQ) was used to assess the physical activity level of the participants. This scale determined how often and for how long the participants had been active at light, moderate, and vigorous intensities during the last 7 days. The intensities of physical activity were assigned and based on the overall metabolic equivalent of task (MET) value on the average MET value for each intensity in the MET compendium (Ainsworth et al., 1993, 2000), (3) and a 19-item questionnaire measures the attitudes toward social media, reasons toward using social media platforms, and favorite social network platforms in self-quarantine during the COVID-19 outbreak. The survey instrument applied a five-point Likert scale (1 = strongly disagree and 5 = strongly agree). A draft questionnaire was piloted and revised to a final survey related to electronic survey response and demographic variables, which took approximately 15 min to complete. The instrument was reviewed by three academic specialists in public health, physical activity, and social media to validate the instrument items. The specialists approved all instrument items after the researcher eliminated all non-approved items. A reliability test that has been established by Cronbach's alpha also was used to examine the stability of measurement and ensure that there is no conflict between the outlines of the survey. The test result approved the reliability of the model of the instrument with a value of 0.969. All the responses of the participants were recorded *via* a platform of the study survey and downloaded by a trained researcher.

### Statistical Analysis

Analysis was carried out using Statistical Package for the Social Sciences (SPSS) (version 23.0). Microsoft Excel was used for data entry, editing, and sorting. Continuous data were presented as mean and SD, and categorical data as frequency and percentage. A multiple linear regression analysis was used to assess the predictors associated with attitudes toward the use of social media and the demographic characteristics of participants. Statistical significance was set at *P* < 0.05.

## Results

A total of 323 physically active individuals participated in this study. The demographic characteristics of the participants were presented in Table 1. Of these 323 participants, 29.1% were in the age group of 18–24 years, 66.6% were women, and 67.8% were single. More than half of the sample had a bachelor's degree (60.7%) and had a normal BMI level (63.1%). The majority of the participants were living in the middle or central region (83%). The proportion of participants whose METs-min/week from vigorous activity <1,680 was 31.9%, and all of the participants follow people or pages in social media related to sports teams and fitness models. The average number of hours spent on social media per day was 2.95 ± 0.90 h.

**Table 1:** Demographic characteristic of the participants.

**Variable**		***N* = 323 (%)**
Age	18–24	94 (29.1)
	25–29	79 (24.5)
	30–34	56 (17.3)
	35–39	53 (16.4)
	40 and above	41 (12.7)
Gender		
	Male	108 (33.4)
	Female	215 (66.6)
Marital status		
	Single	219 (67.8)
	Married	104 (32.2)
Educational level		
	Less than high school	2 (0.6)
	High school/diploma	60 (18.6)
	Bachelor's degree	196 (60.7)
	High education degree	65 (20.1)
Region		
	Middle region	268 (83)
	Eastern and Western region	47 (14.5)
	Northern and Southern Western	8 (2.5)
BMI		
	Normal	204 (63.1)
	Overweight	90 (27.9)
	Obese	29 (9)
Level of physical activity		
	METs-min/week from moderate activity <1,680	222 (68.1)
	METs-min/week from vigorous activity >1,680 METs-min/week	103 (31.9)
Average hours spent on social media a day	M = 2.95 (SD 0.90)	
People/Pages you follow on social media	Sport teams/Fitness models	323 (100)
	Health/nutrition	171 (52.9)
	Celebrities	125 (38.6)
	News	175 (54.1)

**Figure 1:**
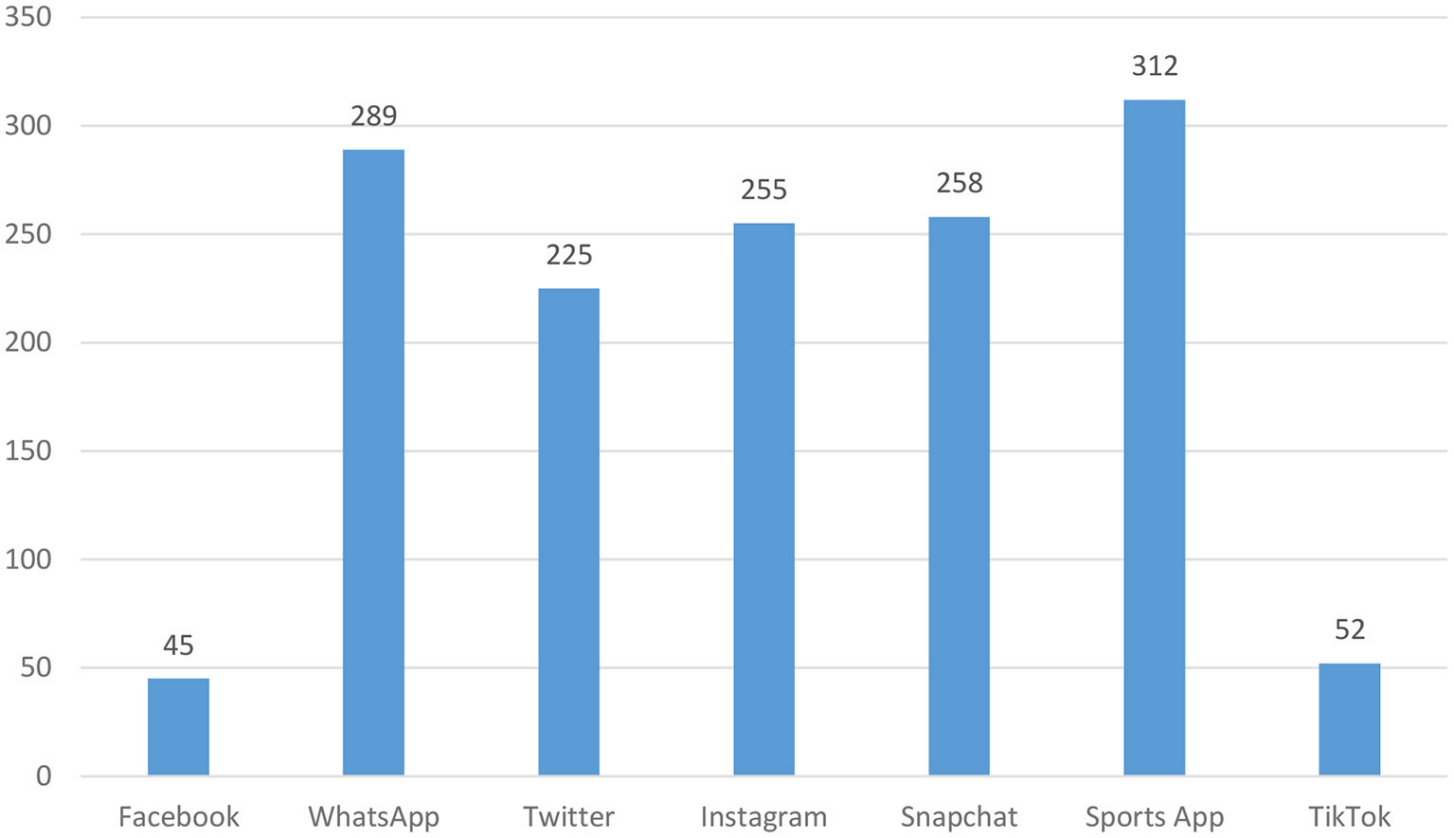
Social media application used by the participants.

Table 2 shows the item and item response in the attitude scale toward social media. The frequency of the most widely used social media application used by the participants is shown in Figure 1.

**Table 2:** The frequencies of participants in terms of their attitudes toward social media.

**Items**	**Strongly disagree**	**Disagree**	**Neutral**	**Agree**	**Strongly Agree**
**During quarantine, I use social media**.					
To stay in touch with friends.	1 (0.3)	8 (2.5)	23 (7.1)	135 (41.8)	156 (48.3)
To keep in touch with relatives.	6 (1.39)	29 (9.0)	36 (11.1)	118 (36.5)	134 (41.5)
Because my friends do.	13 (4)	58 (18)	56 (17.3)	99 (30.7)	97 (30)
To get more followers and friends.	43 (13.3)	111 (34.4)	90 (27.9)	50 (15.5)	29 (9)
To find information about celebrities and keep track of them.	62 (19.2)	107 (33.1)	83 (25.7)	49 (15.2)	22 (6.8)
To pass time when bored.	2 (0.6)	18 (5.6)	41 (12.7)	159 (49.2)	103 (31.9)
To motivate me to participate in physical activities.	4 (1.2)	24 (7.4)	51 (15.8)	120 (37.2)	124 (38.4)
To join sport competition.	21 (6.5)	69 (21.4)	62 (19.2)	93 (28.8)	78 (24.1)
To store and organize contact information (such as email addresses, birthdays and appointments).	22 (6.8)	55 (17)	80 (24.8)	103 (31.9)	63 (19.5)
To listen to music.	16 (5)	28 (8.7)	44 (13.6)	105 (32.5)	130 (40.2)
To read funny text (jokes, riddles, stories, etc.).	8 (2.5)	36 (11.1)	58 (18)	120 (37.2)	101 (31.3)
To share videos and images.	4 (1.2)	32 (9.9)	59 (18.3)	130 (40.2)	98 (30.3)
To express my views on a subject.	22 (6.8)	47 (14.6)	66 (20.4)	110 (34.1)	78 (24.1)
To access activities for educational purposes.	8 (2.5)	24 (7.4)	48 (14.9)	126 (39)	117 (36.2)
	**Almost never**	**Never**	**Sometimes**	**Always**	**Almost always**
**During quarantine…**					
I struggle to stay in places where I will not have access to social media sites.	16 (5.0)	63 (19.5)	88 (27.2)	107 (33.1)	49 (15.2)
My relatives and friends complain that I spend too much time using social media sites.	38 (11.8)	131 (40.6)	71 (22)	55 (17.0)	28 (8.7)
I feel guilty for the time I spend on social media sites.	11 (3.4)	85 (26.3)	87 (26.9)	88 (27.2)	52 (16.1)
I lose track of time when I use social media sites.	6 (1.9)	22 (6.8)	56 (17.3)	154 (47.7)	85 (26.3)

Multiple regression analysis was used to determine the predictors associated between general characteristics and attitudes toward social media (Table 3). Eight variables were included in the analysis (age, gender, marital status, educational level, region, BMI level, physical activity level, and average of hours spent on social media a day). Of the eight variables, age, level of physical activity, and the average of hours spent on social media emerged as significant predictors (*P* < 0.05). The analysis shows that younger individuals showed poorer attitudes than their counterparts. Conversely, those physically active individuals who had METs-min/week from vigorous activity >1,680 had a more positive attitude than those who had <1,680 METs-min/week from vigorous activity (*P* < 0.05).

**Table 3:** Predictors associated with attitudes of physically active individuals toward the use of social media.

**Variable**	***B***	**SE**	***t***	**95% (CI)**	***P***
Age	−1.12	0.49	−2.28	(−2.08 to −0.15)	**0.023** ^*^
Gender	−1.75	1.05	−1.66	(−3.82 to 0.31)	0.096
Marital status	2.89	1.39	2.07	(−2.08 to −0.15)	0.069
Educational level	0.16	0.78	0.21	(−1.36 to 1.70)	0.829
Region	−1.40	0.62	−2.24	(−2.63 to −0.17)	0.076
BMI	−1.20	0.76	−1.57	(−2.70–0.300)	0.116
Level of physical activity	7.30	0.88	8.27	(5.57–9.04)	**0.001** ^*^
Average hours spent on social media a day	2.32	0.51	4.48	(1.30–3.35)	**0.001** ^*^

*CI, confidence interval*;

* *Significant at 0.05*.

## Discussion

The findings of this study provided a baseline finding of the level of physical activity of physically active individuals during the COVID-19 pandemic in Saudi Arabia. The results identified that only 30% of participants had METs-min/week from vigorous activity. Our findings were parallel to a study conducted in France in which there were differences in levels of vigorous activities during the COVID-19 pandemic (Marchant et al., 2021). However, the findings contraindicated the results of the previous study showing the negative effect of home quarantine on physical activity among adults in Saudi Arabia (Alfawaz et al., 2021). A previous study shows a significant increase in physical activity including swimming and physical activity with weights during home quarantine (Alfawaz et al., 2021). The reported low number of participants who did vigorous activity during quarantine was most likely due to the dependence of their routine on gyms to keep them physically fit.

This study also highlights the positive attitudes of physically active individuals toward using social media for exercise and physical activity. The impact of social media usage on physical activity was reported in the previous study (Ammar et al., 2020; Kaur et al., 2020). Previous studies found social media as an effective tool to keep the participants up to date and connected with people to assist with their physical activities (Ammar et al., 2020; Kaur et al., 2020). Despite the positive attitudes found in this study and the impact of social media presented by different studies (Ammar et al., 2020; Kaur et al., 2020), the current results showed that with home activities the level of physical activity is not enough to meet their regular physical activity patterns. Such factors should be considered while formulating and endorsing physical activity programs during the COVID-19 pandemic.

The present survey also revealed that levels of physical activity are significantly associated with attitudes toward social media use. In addition, the results also identified age, level of physical activity, and the average of hours spent on social media emerged as significant predictors of attitudes toward social media use. The results are in line with the findings of the previous study, which showed a significant association between frequencies of social media use and physical activity (Shimoga et al., 2019). However, this is contrary to the findings of the social media use of students and their level of physical activity (Martin et al., 2017). The association of physical activity and social media use among the participants may be due to sharing of activities and feed and related news about exercise. Although there are inconsistencies with the association of physical activity and social media use, it would be beneficial to capitalize on this trend to promote specific interventions and promote healthy behaviors.

The findings of this study have several implications for physically active individuals. The current results show that there may have been changes in the pattern of physical activity of physically active individuals in Saudi Arabia during the COVID-19 pandemic. The positive attitudes of the participants toward social media use may provide benefits in developing physical activity interventions. While the findings may cause concern, it also indicates some opportunities. Hence, the development of physical activity programs during this quarantine and future studies can be based on social media platforms to promote an active healthy lifestyle.

The authors acknowledge some limitations. First, this study is limited in sample size, which may not be generalizable to all physically active individuals who suffer from confinement during the COVID-19 outbreak in Saudi Arabia. Second, the cross-sectional design of the study cannot claim the causality between behaviors toward social media use and physical activity. Finally, the methods in data gathering and the instruments used in this study were limited, which may result in some bias in the study. However, this study offers vital information that can be used as a baseline finding since there is a scarcity of studies addressing attitudes toward social media use and physical activity of physically active individuals in Saudi Arabia.

In conclusion, the present survey results indicate adverse consequences of home quarantine as reflected by a small proportion of participants who had differences in levels of vigorous activities during the COVID-19 pandemic in Saudi Arabia. Future studies and program development for physical activity to maintain an active lifestyle during lockdowns may utilize social media platforms such as fitness apps. Social media appears to be a key avenue to promote and disseminate health interventions to promote physical activity during this COVID-19 pandemic.

## Data Availability Statement

The datasets presented in this article are not readily available because it is locked and stored in the College of Applied Medical Science at King Saud University. The datasets can be obtained from the author on reasonable request.

## Ethics Statement

The studies involving human participants were reviewed and approved by The Institutional Review Board Committee at King Saud University approved the study prior to enrollment in this study. The patients/participants provided their written informed consent to participate in this study.

## Author Contributions

MA contributed to data analysis, interpretation of results, drafting, or revising the manuscript, agreed to be accountable for all aspects of the study, and approved the final version of the manuscript.

## Conflict of Interest

The author declares that the research was conducted in the absence of any commercial or financial relationships that could be construed as a potential conflict of interest.

## Publisher's Note

All claims expressed in this article are solely those of the authors and do not necessarily represent those of their affiliated organizations, or those of the publisher, the editors and the reviewers. Any product that may be evaluated in this article, or claim that may be made by its manufacturer, is not guaranteed or endorsed by the publisher.
